# Robotic investigation on effect of stretch reflex and crossed inhibitory response on bipedal hopping

**DOI:** 10.1098/rsif.2018.0024

**Published:** 2018-03-28

**Authors:** Xiangxiao Liu, Andre Rosendo, Shuhei Ikemoto, Masahiro Shimizu, Koh Hosoda

**Affiliations:** Graduate School of Engineering Science, Osaka University, Japan

**Keywords:** stretch reflex, crossed response, musculoskeletal robot, lateral balance, hopping/bouncing

## Abstract

To maintain balance during dynamic locomotion, the effects of proprioceptive sensory feedback control (e.g. reflexive control) should not be ignored because of its simple sensation and fast reaction time. Scientists have identified the pathways of reflexes; however, it is difficult to investigate their effects during locomotion because locomotion is controlled by a complex neural system and current technology does not allow us to change the control pathways in living humans. To understand these effects, we construct a musculoskeletal bipedal robot, which has similar body structure and dynamics to those of a human. By conducting experiments on this robot, we investigate the effects of reflexes (stretch reflex and crossed inhibitory response) on posture during hopping, a simple and representative bouncing gait with complex dynamics. Through over 300 hopping trials, we confirm that both the stretch reflex and crossed response can contribute to reducing the lateral inclination during hopping. These reflexive pathways do not use any prior knowledge of the dynamic information of the body such as its inclination. Beyond improving the understanding of the human neural system, this study provides roboticists with biomimetic ideas for robot locomotion control.

## 1. Introduction

The effects of brain control (e.g. visual and vestibular feedback control) on dynamic locomotion have been widely investigated and recognized [1]. Nevertheless, proprioceptive control, such as reflexive control, should never be ignored as it can immediately react to a simple sensation. This property increases the importance of reflexive control for maintaining balance during dynamic locomotion, which requires fast reactions to avoid falling over. To understand reflexive control, researchers have widely chosen to implement hopping experiments, because hopping is a simple and representative bouncing gait with complex dynamics. Understanding the human reflexive control can help scientists develop rehabilitation strategies for patients suffering from stroke [2,3] and spinal cord injuries [4,5]. In addition, it can can help roboticists develop methods for robot locomotion control [6,7].

Stretch reflex, a well-known example of reflexive control, contracts a muscle in response to its stretching through the muscle spindles. This feedback control network is a simple local feedback control within a muscle. In human hopping, the stretch reflex occurs in the soleus muscles approximately 40 ms after touchdown [8,9]. The duration of the stretch-reflex-induced muscle activity is within 100 ms [8]. The contributions of the stretch reflex to human locomotion have been widely investigated (e.g. walking [10–12], pedalling [13] and running [14,15]). Most past studies on the stretch reflex focused on the motion in the sagittal plane; however, in real-world locomotion, it is necessary to consider all dimensions, including the frontal plane (lateral direction).

In neuroscience, an increasing number of studies on bipedal locomotion have demonstrated the existence of the so-called crossed response [16–20], which is an inhibitory/excitatory interlimb reflexive network passing through the spinal cord from one muscle to another corresponding muscle in the contralateral leg [18,21]. The crossed response between the soleus muscles, a representative pathway of such networks, usually behaves as a crossed inhibitory effect during motor tasks, as it inhibits the activity of the corresponding muscle in the contralateral leg with a short latency (approx. 40 ms) [18,22]. The inhibitory response increases as the afferent feedback input from the ipsilateral muscle increases [18]. Although the effect of crossed inhibitory response has been investigated in human walking [23], more studies are required to confirm that the change in dynamics caused by these pathways contribute to balance during locomotion.

As both the stretch reflex and crossed inhibitory response modify the activities of muscles in bipedal legs, it can be speculated that they influence the posture in the frontal plane during locomotion. For example, when a human lands with lateral inclination in hopping, the soleus muscle in the first touchdown leg (leaning side) is stretched tighter and generates a larger afferent feedback than the soleus muscle in the second touchdown leg. As larger afferent feedback induces a stronger crossed inhibitory effect [18], the muscular activity of the second touchdown leg should be inhibited more strongly by the crossed response than the first touchdown leg. This difference in muscular activity may cause an incorporation of ground reaction force (GRF) between the two legs, thus helping the body reduce lateral inclination. However, because dynamic locomotion is affected by the neural networks, musculoskeleton and environment, it is very difficult to provide a rational explanation for this in the absence of experiments.

In this study, we implement a robotic constructive experiment because it is difficult to fully understand the effects of these reflexes on dynamic locomotion by conventional approaches, such as experiments on humans and simulations. Although experiments on humans can identify neural pathways, it is difficult to clarify their effects, because the effects of other neural and cognitive processes cannot be removed in living animals [6,24]. A simulation also falls short of this target because the body dynamics including touchdown dynamics are very complex and difficult to be well modelled in a visual environment [6]. In recent years, performing experiments on bioinspired robots has been demonstrated to be a powerful approach for understanding human/animal locomotion, and is garnering increasing attention [6,25–27]. Therefore, we built a musculoskeletal robot that has body dynamics similar to a human; in particular, our robot takes precise anatomical details into account, along with the actuation patterns derived from electromyography (EMG) data.

The rest of the paper is organized as follows. First, we introduce the constructive experiment, including the hardware used, the implementation of the reflexive control by artificial muscles, and the experiment protocol to show the effectiveness of the reflexes. Through 382 hopping trials, we demonstrate that the stretch reflex can help in reducing lateral inclination, and a combination of the stretch reflex and crossed response can contribute to the reduction of lateral inclination even further.

## 2. Material and methods

**Figure 1.**
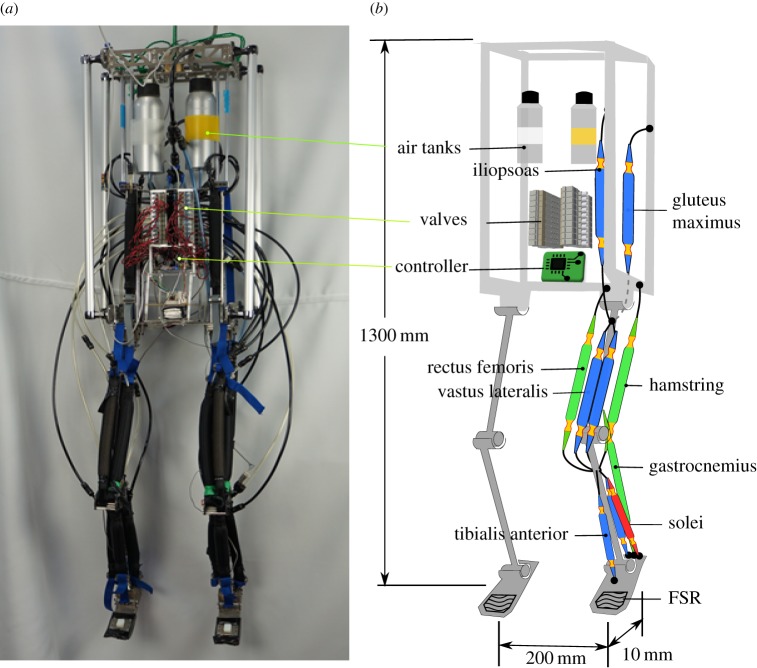
(*a*) Photo of the robot. (*b*) The musculoskeletal robot and its equipments. Monoarticular and biarticular muscles are indicated by blue and green, respectively. The robot has two soleus muscles in each leg: one for generating static force and one for reflexes (red).

Figure 1 shows the musculoskeletal bipedal robot used for the experiment. This robot is built to mimic the human neural networks, muscles and skeleton. It is designed based on the following four ideas.

**Figure 2.**
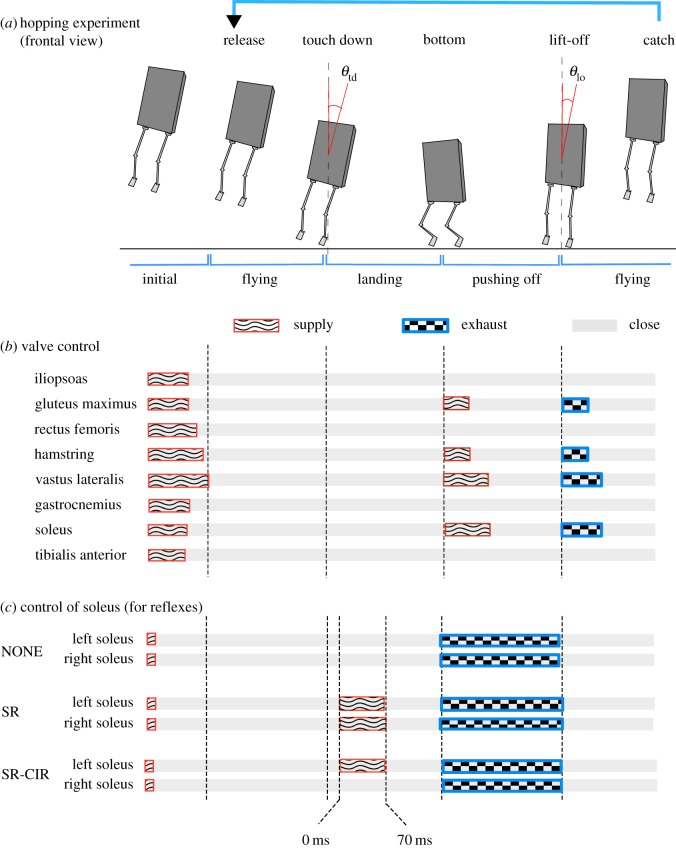
(*a*) Illustration of the hopping experiment. When the robot is released mid-air, it jumps upwards following the predetermined muscle activation. During the hopping, *θ*_td_ and *θ*_lo_ are recorded. (*b*) The valve control for each artificial muscle and (*c*) the valve control of the soleus muscles (for reflexes) when landing with a left inclination. (Online version in colour.)

— Each robot leg has nine representative muscles that imitate the hopping action of a human [28–31]. Soft and elastic pneumatic artificial muscles (PAMs) are used as the actuators of the robot. A PAM contracts when compressed air is supplied, and relaxes when the air inside the muscle is exhausted. The tensile force of a PAM is a function of the deformation and inner air pressure [32].
— The hopping control (figure 2*b*) is based on human EMG data during hopping/jumping [28–31]. It enables the robot to reach a hopping apex of approximately 200 mm when it is released from a height of 200 mm. The duration from the moment when it is released to the next apex is approximately 1 s.
— The centre of mass (COM) was designed to be at 57% of the height of the body when standing (similar to a male human [33]). The height and width of the body are 1330 and 200 mm (distance between both hip joints), respectively. Its weight is 7.8 kg.
— We simplified the ankle, knee and hip as hinge joints, because the main contributions of these joints are within the sagittal plane during hopping [34,35].

Detailed information about the PAM, control and other adopted devices is provided in the appendix.

### 2.1. Reflexive control: stretch reflex and crossed inhibitory response

**Figure 3.**
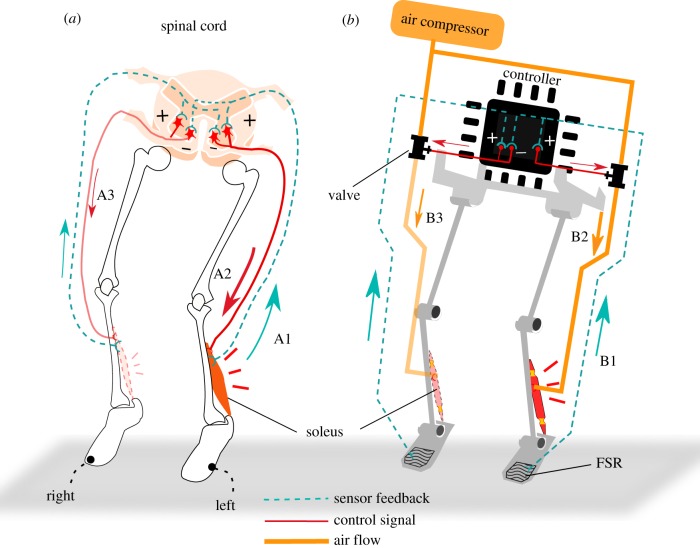
Explanation of stretch reflex and crossed inhibitory response pathways in the case of left-leaning landing. (*a*) In humans, the stretching of the soleus muscle generates afferent feedback (A1). This afferent feedback elicits the activity (stretch reflex) of the ipsilateral soleus muscle (A2). Crossing the spinal cord, the afferent feedback inhibits the soleus muscle activity (crossed inhibitory response) of the contralateral leg (A3). The leaning-side (left) soleus muscle is stretched more and induces a stronger afferent feedback. This triggers a stronger crossed inhibitory response to the right soleus muscle than the crossed inhibitory response from the right to the left. (*b*) In the robot, the FSR sensors detect the stretching of the soleus muscle (B1) and compressed air is supplied to contract the soleus muscle, mimicking the stretch reflex (B2). To replicate the crossed inhibitory response, the stimulation of the FSR in the ipsilateral (left) leg inhibits the air supply to the contralateral soleus muscle (B3).

We qualitatively replicated the stretch reflex and crossed inhibitory response in the soleus muscles of our robot considering the current technology and cost. For the stretch reflex, in humans, a muscle contracts in response to its stretching through the muscle spindles (figure 3*a* A1 to A2). In the robot, the touch sensors called force sensitive resistors (FSRs), are used to detect both the touchdown and beginning of muscular stretching (figure 3*b* B1). The duration of the stretch-reflex-induced muscle activity of a human is within 100 ms [8]; therefore, this is simulated by using an air supply for a duration of 70 ms in the robot (figure 3*b* B2). The latency of this stretch reflex is 17–22 ms, which is the sum of the controller delay and the delay of the pneumatic valves, whereas the stretch reflex latency of a human is approximately 40 ms [8,9].

Crossing the spinal cord, the afferent feedback (figure 3*a* A1) inhibits the soleus muscle activity of the contralateral leg (figure 3*a* A3) [20,36]. This is the crossed inhibitory response in humans. When a human lands with lateral inclination, the soleus muscle of the first touchdown leg (the leaning side) is stretched tighter and generates a larger afferent feedback than the soleus muscle in the second touchdown leg. Because a larger afferent feedback induces a stronger inhibitory effect [18], the first touchdown generates a stronger crossed inhibitory response to the second touchdown leg compared to that generated by the second touchdown to the first (shown in figure 3*a*). Our robot is designed to mimic this cross inhibitory response behaviour qualitatively; the first touchdown signal inhibits the air supply of the contralateral soleus muscle (figure 3*b* B1 to B3), while the second touchdown signal of the contralateral leg does not generate a crossed inhibitory response.

We tested three cases of reflexive control, representing different combinations of the stretch reflex and crossed inhibitory response (shown in table 1). The control of air supply for each case is shown in figure 2*c*.

**Table 1.** Reflexive controls for soleus muscles.^a^

	stretch reflex	crossed inhibitory response
NONE	×	×
SR	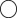	×
SR-CIR	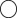	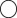

^a^SR, stretch reflex; CIR, crossed inhibitory response.

### 2.2. Experimental methods

In human hopping, there is a small random rotation of the body during the flying phase. Moreover, the height of hopping is not exactly the same, and the terrain is not perfectly flat. Therefore, we have to investigate the effects of reflexive control in these undetermined but possible situations. To simulate such situations, we conducted a large number of experimental trials with a real robot.

The hopping experiments were implemented by dropping the robot from approximately the same height at various initial lateral inclinations. For each hopping trial (figure 2*a*), the experimenter first released the robot from a height of approximately 200 mm in mid-air. When the robot landed on the ground, the valve operations described in figure 2*b* were executed by initiating the FSR triggers, and the robot jumped upwards. Finally, the experimenter grabbed the robot in mid-air. We conducted the aforementioned test for three cases: NONE, SR and SR-CIR. For analysis, the pressure of the soleus muscle (*P*_sol_), lateral inclination at the time of touchdown (*θ*_td_) and lateral inclination at the time of lift-off (*θ*_lo_) during robot hopping were recorded.

The lateral inclination of landing was constrained within ( −6°, 6°). The reason behind this is that if a human lands with a large inclination, it is necessary to change the locomotion pattern to maintain posture, which necessitates the inclusion of other controls such as control from the brain [37].

## 3. Results

**Figure 4.**
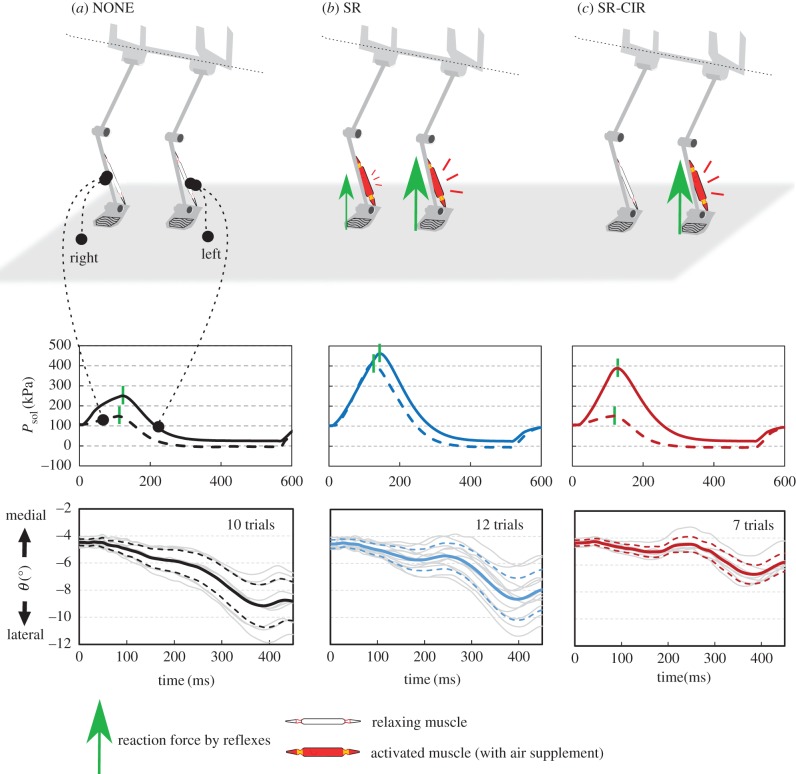
Explanation of posture effects by reflexes during left leaning hopping. Top, muscle activity of each case. Middle, air pressure of the soleus muscles (for reflex) from touchdown to post lift-off. Bottom, lateral inclination (*θ*) from touchdown to lift-off for all trials from −5° < *θ*_td_ < −4°. The selected trials are presented in grey lines. Bold lines indicate the mean and dashed lines indicate the ± standard deviation (s.d.). Landing with an inclination increases the stretch of the soleus muscle in the leaning side and decreases the stretch of the contralateral soleus muscle. In the NONE case (*a*), as the muscles on both sides are supplied with little air, both muscles generate little tension. Thus, the lateral inclination is barely modified. In the SR case (*b*), both soleus muscles are activated. Owing to the lateral inclination, a greater tension is generated by the left soleus muscle and a shifted trend of *θ* is induced. In the SR-CIR case (*c*), due to the crossed inhibitory response, the activity of the soleus muscle in the second touchdown leg is inhibited and a greater shifted trend of *θ* is generated. (Online version in colour.)

To obtain insight into the effects of the reflexes, in figure 4, we demonstrate the representative air pressure of the soleus muscles (for reflex) from touchdown to post lift-off (0–600 ms) and lateral inclination (*θ*) over time during the stance phase with left-leaning landing (−5° < *θ*_td_ < − 4°) trails. Landing with an inclination causes a tighter stretch and higher air pressure in the soleus muscle (*P*_sol_) of the first touchdown leg. In the NONE case, owing to the slight air supply, an insignificant force output is generated by each soleus muscle and the lateral inclination is barely affected. In the SR case, both the soleus muscles are activated. Owing to the inclination, a greater *P*_sol_ (which indicates a greater ground reaction force) is generated by the first touchdown (left) leg and a shifting trend of *θ* is induced. In the SR-CIR case, due to the crossed inhibitory response, the activity of the soleus muscle in the second touchdown leg is inhibited and a greater shift of *θ* is achieved during the stance phase.

**Figure 5.**
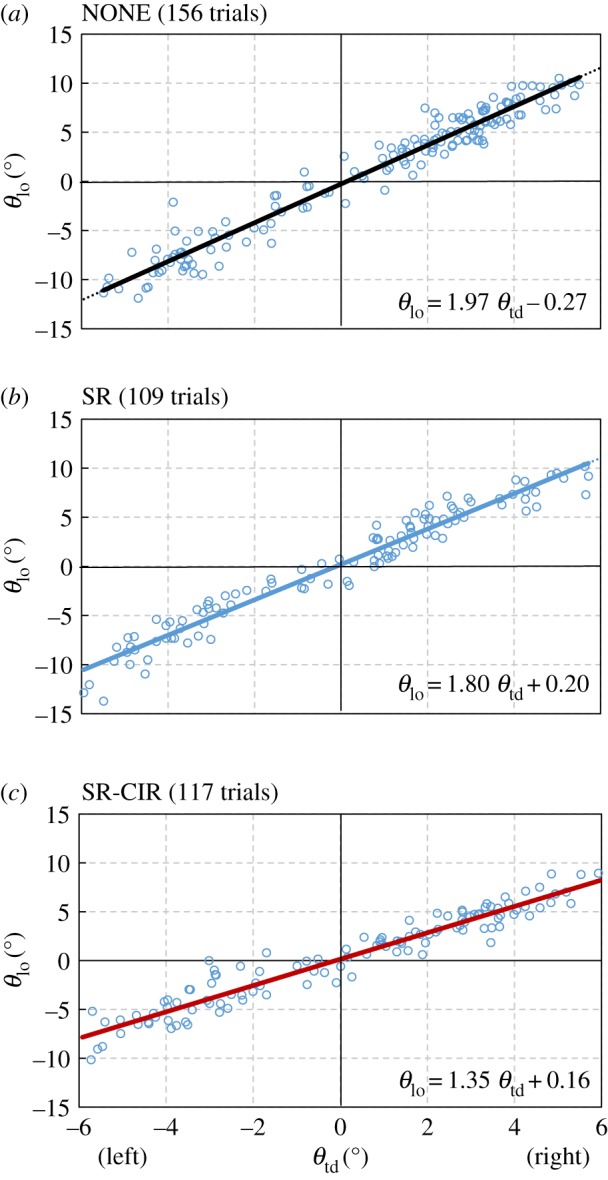
(*a–c*) Results of NONE, SR and SR-CIR cases. For all experiments, one circle represents a hopping trial. The horizontal ordinates indicate the lateral inclination in touchdown (*θ*_td_), and the vertical ordinates represent the lateral inclination in lift-off (*θ*_lo_). Regression lines are presented in each sub-figure. (Online version in colour.)

To evaluate the overall posture effects, figure 5*a*–*c* plot the lateral inclination of both the touchdown (*θ*_td_) and lift-off (*θ*_lo_) for all the trials of the NONE, SR and SR-CIR cases. The number of trials is shown in each case. In each sub-figure, a small circle represents a hopping trial with *θ*_td_ and *θ*_lo_ in the horizontal and vertical ordinates, respectively. Regression lines are presented to evaluate the average performance, because an approximately straight line is generated by the circles in each sub-figure (coefficient of determination: *R*^2^_NONE_ = 0.951, *R*^2^_SR_ = 0.951 and *R*^2^_SR-CIR_ = 0.926). Slope coefficient = 1 indicates that the robot can maintain the lateral inclination after lift-off; a lower value of slope coefficient implies a stronger posture effect. Additionally, as the values of the intercept are small and the regression lines nearly pass through the original point, we will not discuss them in detail.

**Figure 6.**
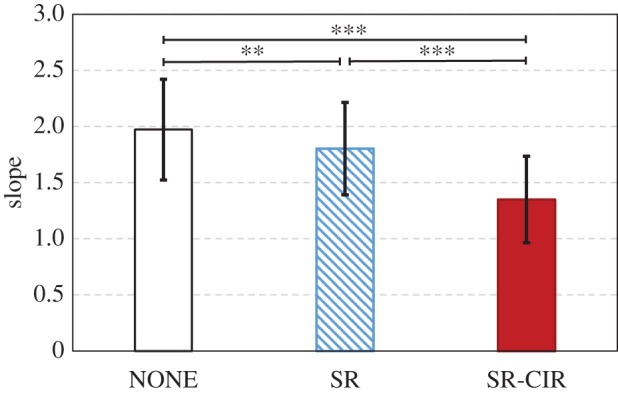
Comparison of the slope coeffecients of the NONE, SR and SR-CIR cases. A smaller slope coefficient indicates a stronger ability to recover the lateral posture. The average slopes of the three cases are significantly different (^**^p < 0.01, and ^***^p < 0.001 after Bonferroni correction). This result shows that: (1) the stretch reflex contributes to reducing the lateral inclination during hopping; and (2) the combination of the stretch reflex and crossed inhibitory response can help reduce the lateral inclination even further. (Online version in colour.)

The slope coefficients are compared in figure 6. Significant differences were observed among the three cases by the analysis of variance (ANOVA) test (*F* = 74.23, *p* < 0.0001). Compared to the NONE case, SR shows a smaller slope and a significant difference (*p* < 0.01, two-tailed unpaired *t*-test after Bonferroni correction). Moreover, SR-CIR exhibits a smaller slope and is significantly different from the SR case (*p* < 0.001, two-tailed unpaired *t*-test after Bonferroni correction). This shows that both the stretch reflex and crossed inhibitory response contribute to decrease the lateral inclination.

## 4. Discussion

Scientists have widely identified the neural pathways in the human body. However, it is difficult for them to clarify the effects of these pathways on locomotion, because current technology does not allow them to change and compare neural pathways in living animals. We aimed to tackle this issue by conducting experiments on a musculoskeletal bipedal robot. In the experiments, we investigated the posture effects induced by both the stretch reflex and crossed inhibitory response during hopping. The results showed that both the stretch reflex and crossed response contribute to reducing the lateral inclination during hopping. The findings in this study can give scientists an insight into understanding the effects of reflexes in dynamic locomotion. Moreover, roboticists can use this study as guidance for developing methods for robot balance control.

In our investigation, although we demonstrated that both the stretch reflex and crossed inhibitory response contribute to the reduction of lateral inclination, even the best scenario (SR-CIR) did not show posture recovery after lift-off. This is reasonable. Because firstly, human locomotion is controlled by numerous muscles. The stretch reflex occurs not only in the soleus muscles but also in other muscles such as vastus lateralis [38] and medial gastrocnemius [9] during hopping. Regarding the crossed response, an increasing number of studies have identified other pathways in the human body [39,40]. As the application of additional reflexive control to other muscles raises the issue of intralimb coordination, in the present experiment, we only focused on the soleus muscles. Moreover, other than reflexes, a human also uses the visual and vestibular systems to maintain his/her locomotion balance [41]. In our study, the influence of other systems was excluded so that we could clarify the effects of the stretch reflex and crossed inhibitory response. In the future, we will investigate the integration of all feedback control systems in our robot.

Previous studies have widely investigated the contributions of the stretch reflex in the sagittal plane [10–15,42,43]. Our results (SR case versus NONE case in figure 6) show that the stretch reflex contributes to the reduction of lateral inclination, and suggest that the stretch reflex contributes to the balance in the frontal plane during hopping. Interestingly, in the SR case, for the landing with either left or right inclination, the applied control is the same (the air supply for the stretch reflex is equal between the two legs). Landing with an inclination induces different amounts of muscle stretch between the two legs (figure 4*a*,*b*). In the SR case, the activated soleus muscle in the leg with tighter stretch (leaning side) generates a GRF than the corresponding muscle in the contralateral leg with weaker stretching (figure 4*b*). Additionally, the muscle with tighter stretching restores and returns more energy during the stance phase. By contrast, in the NONE case (figure 4*a*), both the relaxing soleus muscles react only slightly to the stretch, and therefore the posture is not significantly influenced.

Human experiments have confirmed the crossed responses between the two legs [18,19,22,44–46]. Our result in figure 6 (comparison between SR and SR-CIR) demonstrates that the crossed inhibitory response can significantly contribute to the reduction of the lateral inclination, and implies that it can assist in posture balancing during hopping. This is because the crossed response decreases the activity of the soleus muscle in the second touchdown leg. This induces a large difference in muscular activity between the two legs and can generate a greater force to contribute to posture recovery (figure 4*c*). Moreover, our result corresponds to the recent investigation of crossed response during walking. By comparing the subjects with and without short latency crossed response, Gervasio *et al.* [23] determined that the short latency crossed response can influence the lateral inclination of the body, and suggested that crossed response contributes to the dynamic walking stability.

Considering the similarity between hopping and standing (e.g. bipedal support stance phase), the comparison between the SR case and the NONE case also provides an insight into the observed phenomenon in the experiments on human standing. For example, it was widely observed (this can also be inferred from common sense) that when a human is in unstable/threatening situations (e.g. changes in body orientation [47], standing on a high platform [48] and possibility of support surface change [49,50]), the muscles tend to get facilitated more than when in safe situations. Scientists speculated that this phenomenon may contribute to posture stability [48]. In our research, we demonstrated that equally facilitating the muscles in both legs by the stretch reflex can help in posture balancing (SR case versus NONE case in figure 6) and supported this speculation.

To understand neural networks, scientists widely choose to conduct experiments on animals or use simulations. However, it is difficult to investigate their effects on locomotion through animal experiments, because it is currently impossible to modify and compare neural pathways in living animals. Additionally, issues such as risk of injury and ethics should be considered in such methods. Although such challenges can be overcome by performing simulations, these are not good enough for replicating the compliant interaction between the body and the real environment. Hence it is difficult to investigate locomotion with complex dynamics [6], such as bipedal bouncing. Therefore, in the current study, we constructed a bioinspired musculoskeletal robot. By using this robot, we can modify/investigate neural pathways and conduct hopping experiments in a real environment. Compared to conventional bioinspired robots, our musculoskeletal robot qualitatively improved the level of biometrics. For example, the PAMs can play different roles, such as those of actuators and springs, which are similar to biological muscles [32,51]. In addition, the robot is directly controlled by stimulating the artificial muscles, and the control is based on the observed human muscle activity. Further, we conducted over 300 trials and demonstrated the high durability of our bioinspired robot.

Similar to other robotic studies trying to mimic biological behaviour, our approach has certain limitations. The developed artificial system cannot perfectly replicate the human body. For example, some properties of biological muscles, such as the force–length relationship [42], which can improve the hopping stability, are absent in PAMs [52]. For the stretch reflex and crossed inhibitory response, the magnitude is related to the afferent input in humans [18], whereas its replication in the robot is constant. Furthermore, we used FSRs to detect the start of muscle stretching. Practically, in biological muscles, the stretch is sensed by muscle spindles. Although we are developing artificial muscle spindles to mimic this natural phenomenon [53], the present setting with FSRs would be sufficient to functionally reproduce the stretch reflex and crossed inhibitory response. These issues still need to be resolved in the future.

## Supplementary Material

Response to Referees

## Supplementary Material

Rewording -- a special response to 2nd referee

## Figures and Tables

**Figure 7.**
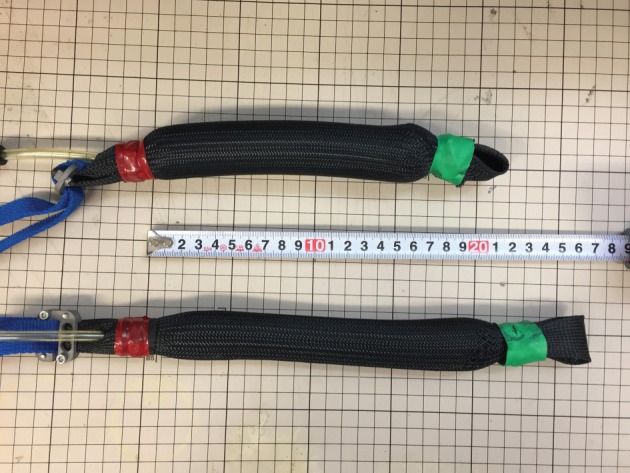
PAMs used in the robot. The upper PAM is supplied with compressed air and the lower PAM is relaxed. The contraction is around 20%. (Online version in colour.)

## Data accessibility

All data created during this research are openly available in the figshare repository (https://doi.org/10.6084/m9.figshare.5943370.v4).

## Appendix A. Pneumatic artificial muscles

PAM is considered as one of the most efficient and widely used artificial muscles [32]. With properties such as elasticity, softness, morphology and high power-weight ratio, we chose PAMs as the robot actuators (samples of PAM are shown in figure 7). By supplying compressed air to a PAM, the PAM can replicate the contraction of a biological muscle. An exhaustion could mimic the muscle relaxation. The force output of a PAM increases with the internal air pressure and deformation (shown in the following equation) [32].

where *F* is the force output, *D*_0_ is the diameter without air supply, *P*_air_ is the internal air pressure and *θ* represents the angle of the braid (a parameter to describe the deformation).

## Appendix B. Musculoskeleton

Based on the observed muscle activity in human hopping, to power the robot achieving jumping, we equipped nine representative muscles in each leg. Those muscles are significantly activated in human hopping. Six of these muscles are monoarticular muscles and three are biarticular muscles. Two soleus muscles are installed in parallel in each leg. One is used as the actuator and the other is used to replicate a stretch reflex. The musculoskeletal structure is shown in figure 1.

## Appendix C. Jumping control

The on–off air valves (VQZ1000 series, SMC Corporation) were used to control the air flow of artificial muscles. The monoarticular muscles were determined to contribute to power generation and the biarticular muscles contribute to the coordination of joints [54,55]. Similar findings in vertical jumping can be found in [29,56]. Consequently, in the motion control, we only activated the monoarticular muscles and kept the air in the biarticular muscles constant. In human hopping, as the gastrocnemius muscle is stretched to nearly its longest length in the bottom position [9], we detected the bottom position by measuring the peak of air pressure in a gastrocnemius muscle (*P*_gas_) (described by the following equation).

Based on the data of human vertical hopping and jumping EMG [29–31] and by considering the past jumping robot control [57,58], we generated the control sequence consisting of three states: flying, landing and pushing off. The control program runs in a loop (shown in figure 2) and explained as follows:

(1) *Flying state:* This is the initialization state for the preparation of the landing. The robot adjusts the air in each muscle to a predetermined initialization pressure. The next state is activated after the first foot touches the ground.
(2) *Landing state:* In this state, except the soleus muscles used to mimic stretch reflex, the robot closes all the valves. A stretch reflex is applied in this state. We applied and tested different reflexive controls on the soleus muscles (for reflexes). This state terminates when the robot reaches the bottom posture in squat.
(3) *Pushing off state:* Compressed air is supplied to gluteus maximus, vastus lateralis and soleus muscles. It generates upward thrust to lift off the robot.

## Appendix D. Other devices

FSRs (FSR-406, Interlink Electronics) with a voltage divider of 7.5 kΩ were used to detect the touchdown and start of muscle stretching. The compressed air was controlled using pneumatic valves (PSE540, SMC Corporation) and generated by a compressor (2000–40 m, Jun Air) through a tether. To measure the air pressure in the PAMs, we used the pressure sensors (PSE530) produced by SMC Corporation. A gyroscope (CRS03, Silicon Sensing Systems) and a three-axis accelerometer (KXR94, Kionix) were combined in a complementary filter to obtain the lateral inclination.

The complementary filter estimated the lateral inclination by using the one-axis data from the gyroscope and two-axis data from the accelerometer. First, the accelerometer information of both *Y* and *Z* axes were used to find the angular projection in the frontal plane:

where *y* and *z* represent the acceleration information in the *Y* and *Z* axes, respectively. *θ*_(*x*,*n*)_ is the lateral inclination (about the *X* axis) calculated by the accelerometer.

The accelerometer shows fast reaction time and large noises, whereas the gyroscope has a more stable output but with larger delay. We estimated the body lateral inclination by combining the calculation of accelerometer and gyroscope. The estimated lateral inclination is presented as follows:

where *w* is the filter weight set as 90 in this experiment and *θ*_g(*x*, *n*)_ is the lateral inclination output of the gyroscope.

Considering that the accelerometer registers angular velocity, the gyroscope used a previous angular estimation to update itself:

where *T* is the sampling duration.
