# Supplementary material for: Robotic investigation on effect of stretch reflex and crossed inhibitory response on bipedal hopping
Source: J R Soc Interface. 2018 Mar 28;15(140):20180024. doi: 10.1098/rsif.2018.0024 (PMC5908536; doi:10.1098/rsif.2018.0024)
Supplement: Rewording -- a special response to 2nd referee [file rsif20180024supp2.pdf]

Robotic Investigation on Effect of Stretch Reflex and Crossed Inhibitory Response on Bipedal Hopping

Xiangxiao Liu[1], Andre Rosendo[2], Shuhei Ikemoto[1], Masahiro Shimizu[1], and Koh Hosoda[1]

Abstract—To maintain ~~the~~ balance ~~in~~ during dynamic locomotion, the effects of proprioceptive sensory feedback control (e.g., reflexive control) ~~can never~~ should not be ignored ~~due to~~ because of its simple sensation and fast reaction. Scientists have identified the pathways of reflexes; however, it is difficult to ~~uncover~~ investigate their effects ~~in~~ during locomotion, because locomotion is controlled by ~~the~~ a complex ~~complicated~~ neural system, and current technology does not allow us to change the control pathways in living humans. To understand ~~the~~ these effects, we construct a musculoskeletal bipedal robot, which has similar body structure and dynamics ~~as that of~~ to a human. By conducting experiments on this robot, we investigate the effects of reflexes (stretch reflex and crossed inhibitory response) on the posture during hopping, a simple and representative bouncing gait with rich dynamics. ~~With~~ Through over 300 hopping trials, we confirm that both the stretch reflex and crossed response can contribute ~~in~~ to reducing the lateral inclination during hopping. These reflexive pathways do not use any prior knowledge of the dynamic information of the body, such as its inclination. Beyond improving the understanding of human neural system, this study provides roboticists with biological ideas ~~on~~ for robot locomotion control.

Note 1

Note 2

Index Terms—stretch reflex, crossed response, musculoskeletal robot, lateral balance, hopping/bouncing

I. Introduction

The effects of brain control (e.g., visual and vestibular feedback control) on dynamic locomotion have been widely investigated ~~and recognized~~ [1]. Nevertheless, ~~the~~ proprioceptive control, such as reflexive control, should never be ignored as ~~they~~ it can immediately react ~~with simple sensation~~ to a simple sensation. This property increases the importance of reflexive control for maintaining balance during dynamic locomotion, which requires fast reactions to avoid falling over. To understand reflexive control, researchers have widely chosen to implement hopping experiments, since hopping is a simple and representative bouncing gait with rich dynamics. Understanding ~~the~~ human reflexive control can help scientists develop rehabilitation strategies for patients ~~with~~ suffering from stroke [2] [3] and spinal cord ~~injury~~ injuries [4] [5]. In addition, it ~~can provide ideas to~~ can help roboticists ~~for developing~~ develop methods for robot locomotion control [6] [7].

Note 3

~~Stretch~~ The stretch reflex, a well-known example of reflexive control, contracts a muscle in response to its stretching through the muscle spindles. This feedback control network is a simple local feedback control within a muscle. In human hopping, the stretch reflex occurs in the soleus muscles approximately 40 ms after touchdown [8] [9]. The duration of the stretch-reflex-induced muscle activity is within 100 ms [8]. The contributions of the stretch reflex ~~in~~ to human locomotion ~~has~~ have been widely investigated (e.g., walking [10] [11] [12], pedaling [13], and running [14] [15]). Most ~~of the~~ past studies on the stretch reflex focused on the motion in the sagittal plane; ~~whereas,~~ however, in ~~real world~~ practical locomotion, it is necessary to consider all ~~the~~ dimensions, including the frontal plane (lateral direction).

Note 4

In neuroscience, an increasing number of studies on bipedal locomotion have ~~shown~~ demonstrated the existence of the ~~so called~~ so-called crossed response [16] [17] [18] [19] [20], which is an inhibitory/excitatory interlimb reflexive network passing through the spinal cord from one muscle to ~~the~~ another corresponding muscle in the contralateral leg [18] [21]. The crossed response between the soleus muscles, a representative pathway of such networks, usually behaves as a crossed inhibition during motor tasks, as it inhibits the activity of the corresponding muscle in the contralateral leg with a short latency (approximately 40 ms) [18] [22]. The inhibitory response increases as the ~~increment of the~~ afferent feedback input from the ipsilateral muscle increases [18]. Although the effect of crossed inhibitory response

Note 5

[1]Department of Engineering Science, Osaka University, Osaka, Japan.
[2]Department of Engineering, University of Cambridge, Cambridge, UK.

has been investigated in human walking [23], more studies are ~~needed~~ required to confirm that the change in dynamics caused by these pathways contribute to ~~the~~ balance ~~in~~ during locomotion.

Since both the stretch reflex and crossed inhibitory response modify the activities of muscles in ~~the~~ bipedal legs, it can be speculated that they influence the posture in the frontal plane during locomotion. For example, when a human lands with lateral inclination in hopping, the soleus muscle in the first touchdown leg (leaning side) is stretched stronger and generates a larger afferent feedback than ~~that of~~ the soleus muscle in the second touchdown leg. Since larger afferent feedback induces a stronger crossed inhibitory effect [18], the muscular activity of the second touchdown leg should be inhibited ~~stronger~~ more strongly by the crossed response ~~compared to that of~~ than the first touchdown leg. This difference in ~~of~~ muscular activity may cause an incorporation of ground reaction force (GRF) between the two legs, thus ~~help~~ helping the body reduce ~~the~~ lateral inclination. ~~While,~~ However, because dynamic locomotion is affected by the neural networks, musculoskeleton, and environment, it is very difficult to ~~make~~ provide a rational explanation ~~with~~ for this in the absence of ~~experiment~~ experiments.

Note 7

In this study, we implement a robotic constructive experiment since it is difficult to fully understand the effects of these reflexes ~~to the~~ on dynamic locomotion by conventional approaches, such as experiments on ~~human~~ humans and ~~simulation~~ simulations. Although experiments on humans can identify ~~the~~ neural pathways, it is difficult ~~for them~~ to clarify ~~the~~ their effects, since ~~they cannot remove~~ the effects of other neural and cognitive processes cannot be removed in living animals [6] [24]. A simulation ~~fall~~ also falls short of this target because the body dynamics, including touchdown dynamics, are very ~~complicated~~ complex and difficult to ~~be well modeled~~ model fully in a visual environment [6]. In recent years, performing experiments on bio-inspired robots has been demonstrated to be a powerful approach for understanding human/animal locomotion, and is ~~gathering~~ ~~increased~~ garnering increasing attention [6] [25] [26] [27]. Therefore, we built a musculoskeletal robot that has body dynamics similar to a human; ~~especially~~ in particular, our robot takes ~~the~~ precise anatomical details into account, ~~together with~~ along with the actuation patterns derived from electromyography (EMG) data.

The rest of the paper is organized as follows. First, we introduce the constructive experiment, including the ~~used~~ hardware used, the implementation of the reflexive control by artificial muscles, and the experiment protocol, to show the effectiveness of the reflexes. ~~With~~ Through 382 hopping trials, we demonstrate that the stretch reflex can help ~~reducing the~~ in reducing lateral inclination, and ~~the~~ a combination of the stretch reflex and crossed response can contribute to the reduction of ~~the~~ lateral inclination even further.

Note 6

II. Methods

Fig. 1 shows the musculoskeletal bipedal robot used for the experiment. This robot is built to mimic the human neural networks, muscles, and skeleton. It is designed based on the following four ideas:

- Each robot leg has nine representative muscles that imitate the hopping action of a human [28] [29] [30] [31]. Soft and elastic pneumatic artificial muscles (PAMs) are used as the actuators of the robot. A PAM contracts when compressed air is supplied, and relaxes when the air inside the muscle is exhausted. The tensile force of a PAM is a function of the deformation and inner air pressure [32].
- The hopping control (Fig. 2 (b)) is based on ~~the~~ human electromyographic (EMG) data during hopping/jumping [29] [30] [31] [28]. It enables the robot to reach a hopping apex of approximately 200 mm when it is released from a height of 200 mm. The duration from the moment when it is released to the next apex is approximately 1 s.
- The centre of mass (COM) was designed to be at 57% of the height of the body, ~~in~~ when standing (similar to a male human [33]). The height and width of the body are 1330 mm and 200 mm (distance between both hip joints), respectively. Its weight is 7.8 kg.
- We simplified the ankle, knee, and hip as hinge joints, because the main contributions of these joints are within the sagittal plane during hopping [34] [35].

Detailed information about the PAM, control, and other adopted devices ~~are~~ is provided in the Appendix.

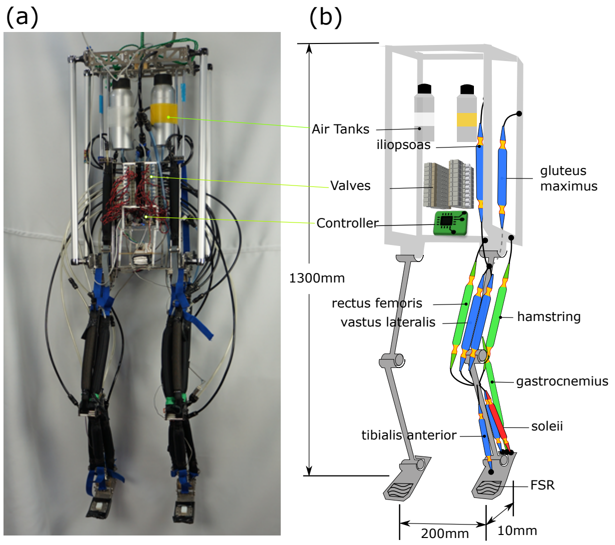

Fig. 1. (a) ~~Photo~~ Photograph of the robot. (b) The musculoskeletal robot and its ~~equipments.~~ equipment. Monoarticular and biarticular muscles are indicated by blue and green, respectively. The robot has two soleus muscles in each leg: one for generating static force and one for reflexes (red).

A. Reflexive control: Stretch ~~Reflex and Crossed Inhibitory Response~~ reflex and crossed inhibitory response

We qualitatively replicated the stretch reflex and crossed inhibitory response in the soleus muscles of our robot, considering the current technology and cost. For the stretch reflex, in ~~human~~ humans, a muscle contracts in ~~responding~~ response to its stretching through the muscle spindles (Fig. 3(a) A1 to A2). In the robot, the touch sensors, called force sensitive resistors (FSRs), are used to detect both the touchdown and beginning of muscular stretching (Fig. 3(b) B1). The duration of the stretch-reflex-induced muscle activity of a human is within 100 ms [8]; therefore, this is simulated by using an air supply for a duration of 70 ms in the robot (Fig. 3(b) B2). The latency of this stretch reflex is 17-22 ms, which is the sum of the controller delay and the delay of the pneumatic valves, whereas the stretch reflex latency of a human is approximately 40 ms [8] [9].

Crossing the spinal cord, the afferent feedback (Fig. 3(a) A1) inhibits the soleus muscle activity of the contralateral leg (Fig. 3(a) A3) [36] [20]. This is the crossed inhibitory response in ~~human.~~ humans. When a human lands with lateral inclination, the soleus muscle of the first touchdown leg (the leaning side) is stretched stronger and generates a larger afferent feedback ~~compared to that of~~ than the soleus muscle in the second touchdown leg. Because a larger afferent feedback induces a stronger inhibitory effect [18], the first touchdown generates a stronger crossed inhibitory response to the second touchdown leg compared to that generated by the second touchdown leg to the first (shown in Fig. 3(a)). Our robot is designed to mimic this cross inhibitory response behaviour qualitatively; the first touchdown signal inhibits the air supply of the contralateral soleus muscle (Fig. 3(b) B1 to B3), while the second touchdown signal of the contralateral leg does not generate a crossed inhibitory response.

We tested three cases of reflexive control, representing different combinations of the stretch reflex and crossed inhibitory response (shown in Table I). The control of air supply for each case is shown in Fig. 2(c).

TABLE I
REFLEXIVE CONTROLS FOR SOLEUS MUSCLES[3]

	Stretch Reflex	Crossed Inhibitory Response
NONE	×	×
SR	○	×
SR-CIR	○	○

[3]SR = Stretch Reflex, CIR = Crossed Inhibitory Response

B. Experimental methods

In human hopping, there is a small random rotation of the body during the flying phase. Moreover, the height of hopping is not exactly the same, and the terrain is not perfectly flat, ~~as well~~. Therefore, we have to investigate the effects of ~~the~~ reflexive control in these undetermined but possible situations. To ~~emulate~~ simulate such situations, we conducted a large number of experimental trials with a real robot.

The hopping experiments were implemented by dropping the robot from ~~almost~~ approximately the same height at various initial lateral inclinations. For each hopping trial (Fig. 2(a)), the experimenter first released the robot ~~in mid-air~~ from a height of around 200 mm in mid-air. When the robot landed on the ground, the valve operations described in Fig. 2(b) were executed by initiating the FSR triggers, and the robot jumped ~~up~~ upwards. Finally, the experimenter grabbed the robot in mid-air. We conducted the aforementioned test for three cases: NONE, SR, and SR-CIR. For analysis, the pressure of the soleus muscle (P_{sol}),

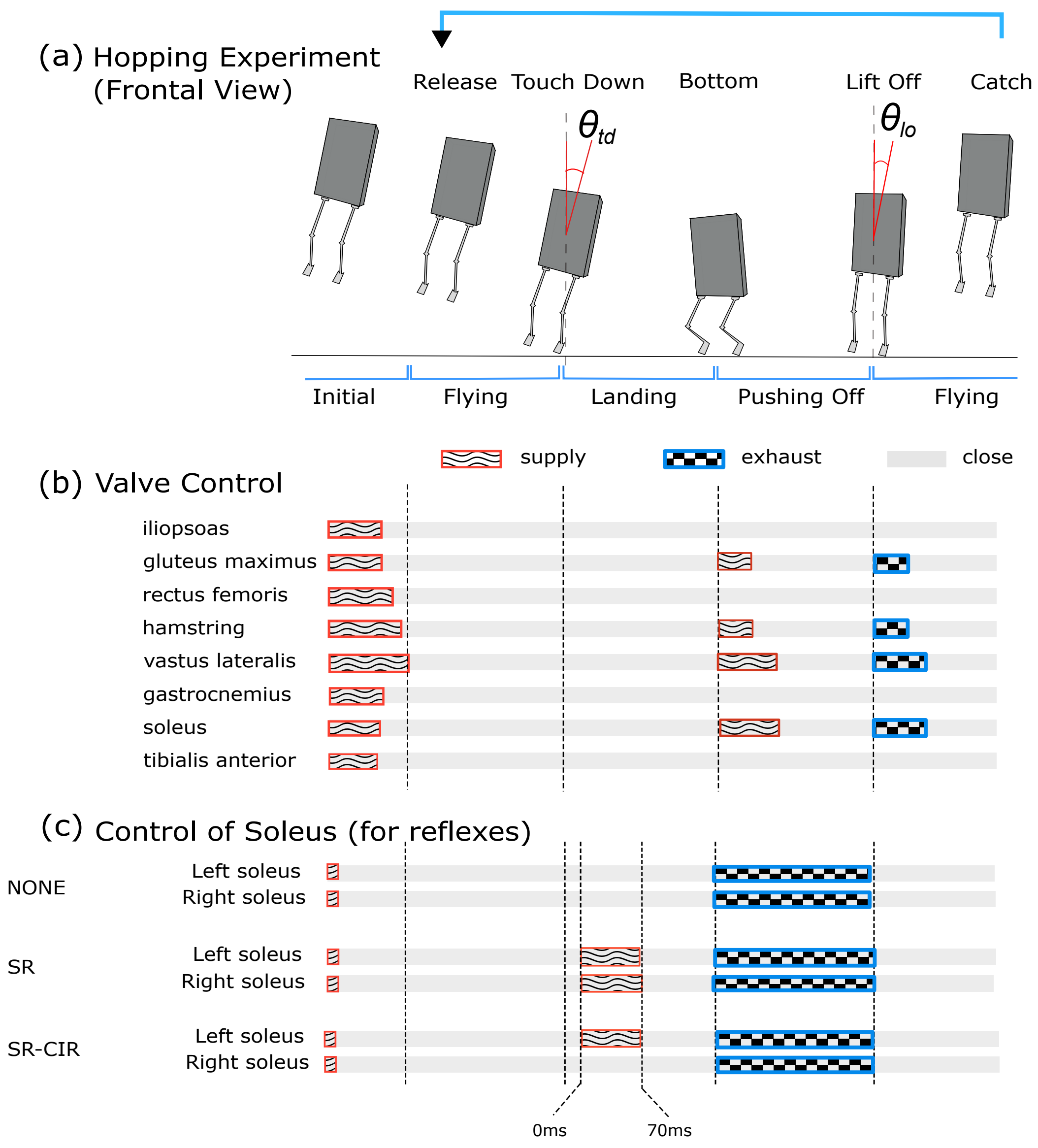

Fig. 2. (a) Illustration of the hopping experiment. When the robot is released mid-air, it jumps up following the predetermined muscle activation. During the hopping, θ_{td} and θ_{lo} are recorded. (b) The valve control for each artificial muscle and (c) the valve control of the soleus muscles (for reflexes) when landing with a left inclination.

lateral inclination at the time of touchdown (θ_{td}), and lateral inclination at the time of lift-off (θ_{lo}) during robot hopping were recorded.

The lateral inclination of landing was constrained within $(-6°, 6°)$. The reason behind this is that if a human lands with a large inclination, it is necessary to change the locomotion pattern to maintain the posture, which needs to include other controls such as the control from brain [37].

III. Results

To get an insight into the effects of the reflexes, in Fig. 4, we demonstrate the representative air pressure of the soleus muscles (for reflex) from touchdown to post lift-off (0 – 600 ms) and lateral inclination (θ) over time during the stance phase with left-leaning landing ($-5° < \theta_{td} < -4°$) trails. Landing with an inclination causes a stronger stretch and higher air pressure in the soleus muscle (P_{sol}) of the first touchdown leg. In the NONE case, owing to the slight air supply, an insignificant force output is generated by each

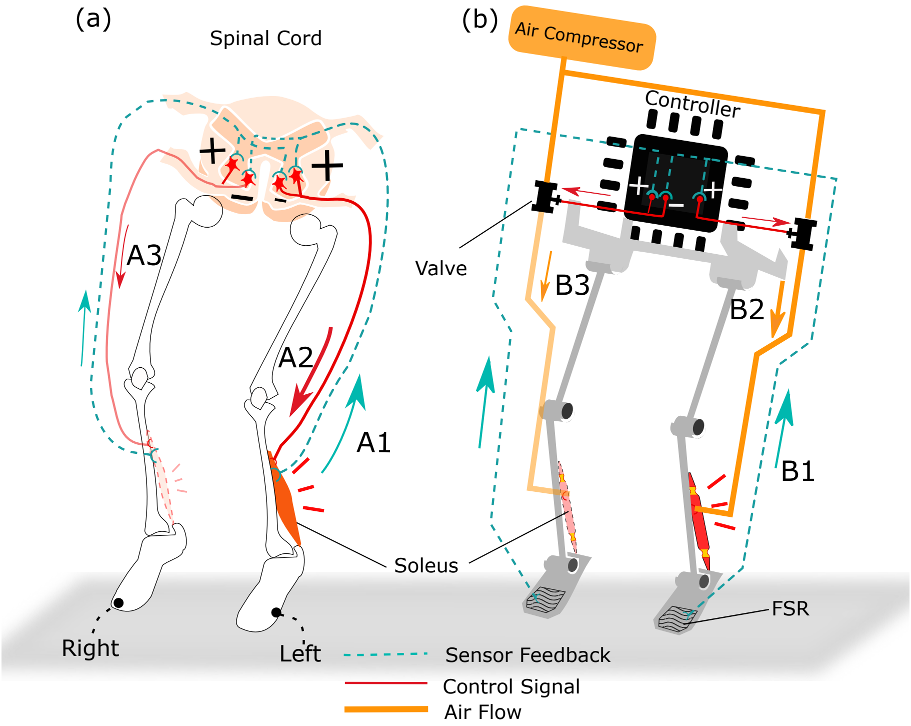

Fig. 3. Explanation of stretch reflex and crossed inhibitory response pathways ~~with a~~ in the case of left-leaning landing. (a) In humans, the stretching of the soleus muscle generates afferent feedback (A1). This afferent feedback elicits the the activity (stretch reflex) of the ipsilateral soleus muscle (A2). Crossing the spinal cord, the afferent feedback inhibits the soleus muscle activity (crossed inhibitory response) of the contralateral leg (A3). The leaning-side (left) soleus muscle ~~gets a lager stretch~~ is stretched more and induces a stronger afferent feedback. This triggers a stronger crossed inhibitory response to the right soleus muscle than the crossed inhibitory response from the right to the left. (b) In the robot, the FSR sensors detect the stretching of the soleus muscle (B1), and compressed air is supplied to contract the soleus muscle, mimicking the stretch reflex (B2). To replicate the crossed inhibitory response, the stimulation of the FSR in the ipsilateral (left) leg inhibits the air supply to the contralateral soleus muscle (B3).

soleus muscle, and the lateral inclination is barely affected. In the SR case, both the soleus muscles are activated. Due to the inclination, a greater P_{sol} (which indicates a greater ground reaction force) is generated by the first touchdown (left) leg, and a shifting trend of θ is induced. In the SR-CIR case, due to the crossed inhibitory response, the activity of the soleus muscle in the second touchdown leg is inhibited, and a greater shift of θ is achieved during the stance phase.

In order to evaluate the overall posture effects, Figs. 5(a)–(c) plot the lateral inclination of both the touchdown (θ_{td}) and lift-off (θ_{lo}) for all the trials of the NONE, SR, and SR-CIR cases. The number of trials is shown in each case. In each sub-figure, a small circle represents a hopping trial with θ_{td} and θ_{lo} in the horizontal and vertical ordinates, respectively. Regression lines are presented to evaluate the average performance, since an ~~approximate~~ approximately straight line is generated by the circles in each sub-figure (coefficient of determination: $R^2_{NONE} = 0.951$, $R^2_{SR} = 0.951$, and $R^2_{SR-CIR} = 0.926$). Slope coefficient $= 1$ indicates that the robot can maintain the lateral inclination after lift-off; a lower value of slope coefficient implies a stronger posture effect. Additionally, since the values of the intercept are small and the regression lines nearly pass through the original point, we will not discuss them in detail.

The slope coefficients are compared in Fig. 6. Significant differences were observed among the three cases by the analysis of variance (ANOVA) test ($F = 74.23, P < 0.0001$). Compared to the NONE case, SR shows a smaller slope and a significant difference ($P < 0.01$, two-tailed unpaired t-test after Bonferroni correction). Note 9 Moreover, SR-CIR exhibits a smaller slope and is significantly different from the SR case ($P < 0.001$, two-tailed unpaired t-test after Bonferroni correction). This shows that both the stretch reflex and crossed inhibitory response contribute ~~in~~ to decreasing the lateral inclination.

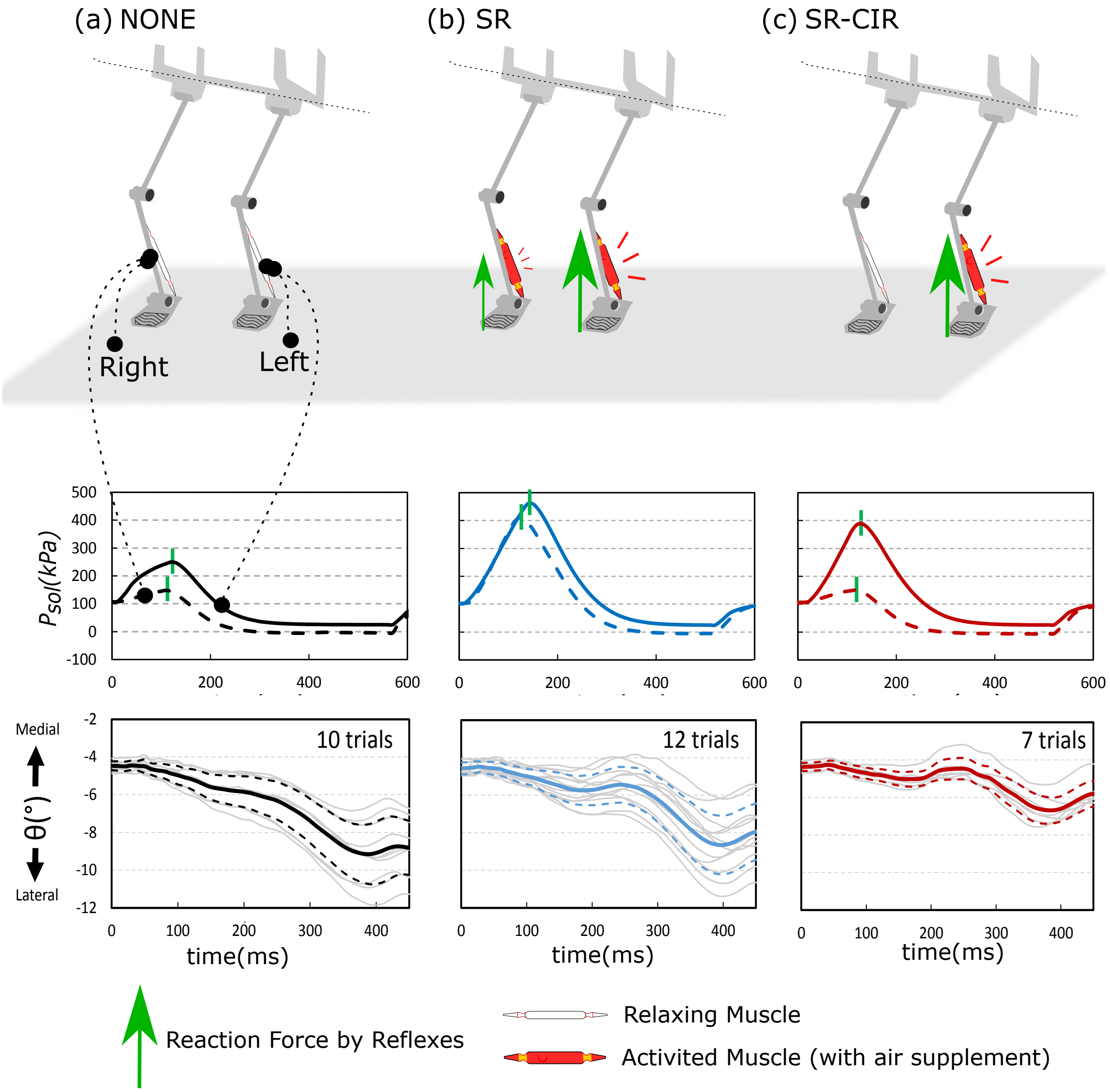

Fig. 4. Explanation of posture effects by reflexes during left leaning hopping. Upper: Muscle activity of each case. Medial: Air pressure of the soleus muscles (for reflex) from touchdown to post lift-off, Lower: Lateral inclination (θ) from touchdown to lift-off for all ~~the~~ trials from $-5^{\circ} < \theta_{lo} < -4^{\circ}$. The selected trials are presented in ~~gray~~ grey lines. Bold lines indicate the mean, and dashed lines indicate the $\pm$ standard deviation (SD). Landing with an inclination increases the stretch of the soleus muscle in the leaning side and decreases the stretch of the contralateral soleus muscle. In the (a) NONE case, since the muscles on both sides are supplied with little air, both muscles generate little tension. Thus, the lateral inclination is barely modified. In the (b) SR case, both soleus muscles are activated. Due to the lateral inclination, a greater tension is generated by the left soleus muscle and a shifted trend of θ is induced. In the SR case, due to the crossed inhibitory response, the activity of soleus muscle in the second touchdown leg is inhibited and a greater shifted trend of θ is generated.

IV. Discussion

Scientists have widely identified the neural pathways in the human body. However, it is difficult for them to clarify the effects of ~~those~~ these pathways on locomotion, because ~~the~~ current technology does not allow them to change and compare ~~the~~ neural pathways in living animals. We ~~challenged~~ aimed to tackle this issue by conducting experiments on a musculoskeletal bipedal robot. In the experiments, we investigated the posture effects induced by both the stretch reflex and crossed inhibitory response during hopping. The results showed that both the stretch reflex and crossed response contribute ~~in~~ to reducing the lateral inclination during hopping. The findings in this study can give scientists an insight ~~to understand~~ into understanding the effects of ~~reflex~~ reflexes in dynamic locomotion. ~~Moreover, roboticists can get ideas from this study on robot balance control.~~ Moreover, roboticists can use this study as guidance for developing methods for robot balance control.

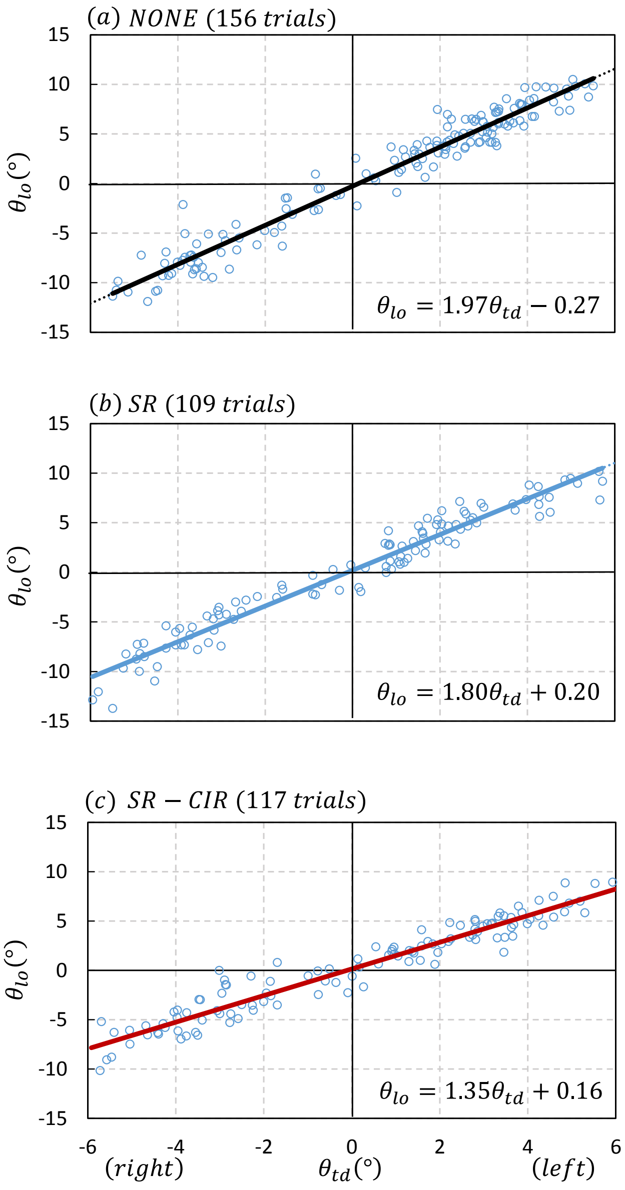

Fig. 5. Results of NONE, SR, and SR-CIR cases. For all experiments, one circle represents a hopping trial. The horizontal ordinates indicate the lateral inclination during ~~in~~ touchdown (θ_{td}), and the vertical ordinates represent the lateral inclination during ~~in~~ lift-off (θ_{lo}). Regression lines are presented in each sub-figure.

In our investigation, although we demonstrated that both the stretch reflex and crossed inhibitory response contribute to the reduction of lateral inclination, even the best scenario (SR-CIR) did not show posture recovery after lift-off. This is reasonable, because, firstly, ~~First,~~ human locomotion is controlled by numerous muscles. The stretch ~~Stretch~~ reflex occurs not only in the soleus muscles, but also in other muscles such as vastus lateralis [38] and medial gastrocnemius [9] during hopping. Regarding the crossed response, an increasing number of studies have identified other pathways in the human body [39] [40]. Since the application of additional reflexive control to other muscles raises the issue of intralimb coordination, in the present experiment, we only focused on the soleus muscles. Moreover, other than ~~except~~ reflexes, a human also uses the visual and vestibular systems to maintain his/her locomotion balance [41]. In our study, the influence of other systems was ~~were~~ excluded so that we could ~~can~~ clarify the effects of the stretch reflex and crossed inhibitory response. In the future, we will investigate the integration of all ~~the~~ feedback control systems in ~~on~~ our robot.

Previous studies have widely investigated the contributions of the stretch reflex in the sagittal plane [10] [11] [12] [13] [14] [15] [42] [43]. Our results (SR case vs. ~~VS.~~ NONE case, in Fig. 6) show that the stretch reflex contributes to the reduction of lateral inclination, and suggest that the stretch reflex contributes to the balance in the frontal plane during hopping. Interestingly, in the SR case, for the landing with either left or right inclination, the applied control is the same (the air supply for the stretch reflex is equal between the two legs). Landing with an inclination induces ~~causes~~ different amounts of muscle stretch between the two legs (Fig. 4(a) and (b)). In the SR case, the activated soleus muscle in the leg with stronger stretch (leaning side) generates a greater reaction force (GRF) than the corresponding muscle in the contralateral leg, with weaker stretching (Fig. 4(b)). Additionally, the muscle with stronger stretching restores and returns more energy during the stance phase. In contrast, in the NONE case (Fig. 4(a)), both the relaxing soleus muscles react only slightly to the stretch, and therefore, the posture is not significantly influenced.

Note 6

Human experiments have confirmed the crossed responses between the two legs [18] [19] [22] [44] [45] [46]. Our result in Fig. 6 (comparison between SR and SR-CIR) demonstrates that the crossed inhibitory response can significantly contribute to the reduction of the lateral inclination, and implies that it can assist in ~~the~~ posture balancing ~~balance~~ during hopping. This is because the crossed response decreases the activity of the soleus muscle in the second touchdown leg. This induces a large difference in muscular activity between the two legs, and can generate a greater force to contribute to posture

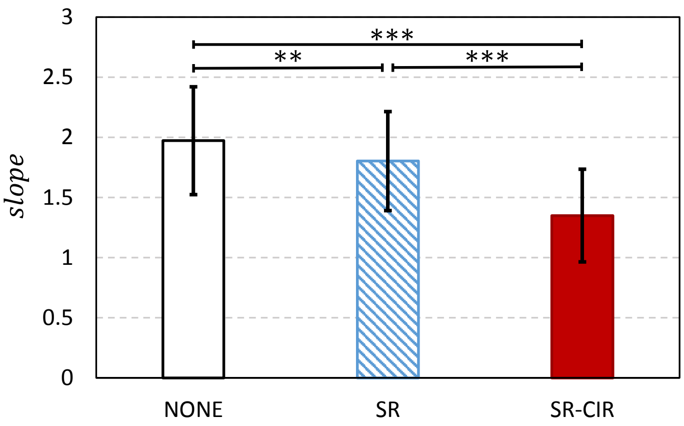

Fig. 6. Comparison of the slope coeffecients of NONE, SR, SR-CIR cases. A smaller slope coefficient indicates a stronger ability to recover the lateral posture. The average slopes of the three cases are significantly different (** $P < 0.01$, and *** $P < 0.001$ after Bonferroni correction). This result shows that: 1) the stretch reflex contribute in reducing the lateral inclination during hopping; and 2) the combination of stretch reflex and crossed inhibitory response can help reducing the lateral inclination even further.

recovery (Fig. 4(c)). Moreover, our result corresponds to the recent investigation of crossed response during walking. By comparing the subjects with and without short latency crossed response, Gervasio et al. [23] determined that the short latency crossed response can influence the lateral inclination of the body, and suggested that crossed response contributes to the dynamic walking stability.

Considering the similarity between hopping and standing (e.g. bipedal support stance phase), the comparison between the SR case and NONE case also provides an insight into understanding the observed phenomenon in the experiments of human standing. For example, it was widely observed (also from common sense) that when a human is in unstable/threatened situations (e.g., changes in body orientation [47], standing on a high platform [48], and possibility of support surface change [49] [50]), the muscles tend to get facilitated stronger compared to that in safe situations. Scientists speculated that this phenomenon may contribute to posture stability [48]. In our research, we demonstrated that equally facilitating the muscles in both legs by stretch reflex can help in posture balance (SR case VS. NONE case in Fig. 6) and supported this speculation.

To understand neural networks, scientists widely choose to conduct animal experiments or simulations. However, it is difficult to investigate their effects in locomotion by animal experiments, since currently it is impossible to modify and compare the neural pathways in living animals. Additionally, issues such as risk of injury and ethics should be considered in such methods. Although such challenges can be overcome by performing simulations, these are not good enough for replicating the compliant interaction between the body and the real environment. Hence it is difficult to investigate locomotion with rich dynamics [6], such as bipedal bouncing. Therefore, in the current study, we constructed a bio-inspired musculoskeletal robot. By using this robot, we can modify/investigate the neural pathways and conduct hopping experiments in a real environment. Compared to conventional bio-inspired robots, our musculoskeletal robot qualitatively improved the level of biometrics. For example, the PAMs can play different roles, such as that of actuators and springs, which are similar to biological muscles [32] [51]. In addition, the robot is directly controlled by stimulating the artificial muscles, and the control is based on the observed human muscle activity. Besides, we conducted over 300 trials and demonstrated the high durability of our bio-inspired robot.

Note 3

Similar to that in other robotic studies trying to mimic biological behaviour, our approach has certain limitations. The developed artificial system cannot perfectly replicate the

~~biological~~ human body. For example, some properties of biological muscles, such as the force-length relationship [42], which can improve the hopping stability, are absent in PAMs [52]. For the stretch reflex and crossed inhibitory response, the magnitude is related to the afferent input in humans [18], whereas its replication ~~on~~ in the robot is constant. Furthermore, we used FSRs to detect the start of muscle stretching. ~~Actually,~~ Practically, in biological muscles, the stretch is sensed by muscle spindles. Although we are developing artificial muscle spindles to mimic this natural phenomenon [53], the present setting with FSRs would be sufficient to functionally reproduce the stretch reflex and crossed inhibitory response. These issues still need to be ~~solved in future~~ resolved in the future.

Author's Contribution

Note 10

X. L. developed the ideas, contributed to the robot development, programing, implemented the experiments, and wrote the manuscript, A. R. contributed to the ideas, robot development, programing, and final manuscript, S, I. contributed to the robot development and final manuscript, M. S. contributed to the final manuscript, K. H. directed the project, contributed to the robot development, contribute to the idea, and contributed to the final manuscript. All authors gave final approval for publication.

Competing Interests

We declare that we have no competing interests.

Funding

This work was supported by JSPS KAKENHI grant number 16J05748, 17H05908, and 23220004.

Acknowledgment

The authors appreciate Prof. Rolf Pfeifer of LIVING WITH ROBOT Ltd./Osaka University for his contributive suggestions/efforts on this research and manuscript. The authors also appreciate Mr. Ahmed Hussain Qureshi, Hirofumi Shin, Arne Hitzmann, and Umakshi Sajnani of Osaka University, for their help during this research. Editage Ltd. helped us improving the English of the manuscript.

APPENDIX A
PAM (PNEUMATIC ARTIFICIAL MUSCLES)

PAM is considered as one of the most efficient and widely used artificial muscles [32]. With properties such as elasticity, softness, morphology, and high power-weight ratio, we chose PAMs as the robot actuators (samples of PAM are shown in Fig. 7). By supplying compressed air to a PAM, the PAM can replicate the contraction of a biological muscle. An exhaustion could mimic the muscle relaxation. The force output of a PAM increases with the internal air pressure and deformation (shown in the following equation) [32].

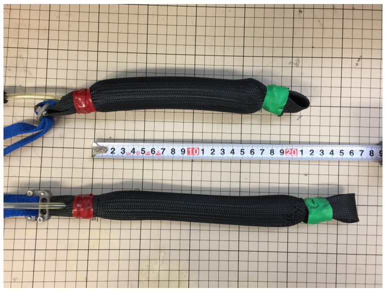

Fig. 7. PAMs used in the robot. The upper PAM is supplied with compressed air and the lower PAM is relaxed. The contraction is around 20%.

$$F = \frac{\pi P_{air} D_0^2}{4}(3\cos^2\theta - 1),$$

where F is the force output, D_0 is the diameter without air supply, P_{air} is the internal air pressure, and θ represents the angle of the braid (a parameter to describe the deformation).

APPENDIX B
MUSCULOSKELETON

Based on the observed muscle activity in human hopping, to power the robot achieving jumping, we equipped nine representative muscles in each leg. Those muscles are significantly activated in human hopping. Six of these muscles are monoarticular muscles and three are biarticular muscles. Two soleus muscles are installed in parallel in each leg. One is used as actuator and the other is used to replicate stretch reflex. The musculoskeletal structure is shown in Fig. 1.

Appendix C
Jumping Control

The on-off air valves (VQZ1000 series, SMC Corporation) were used to control the air flow of artificial muscle. The monoarticular muscles were determined to contribute to power generation and the biarticular muscles contribute to the coordination of joints [54] [55]. Similar findings in vertical jumping can be found in [29] [56]. Consequently, in the motion control, we only activated the monoarticular muscles and kept the air in the biarticular muscles constant. In human hopping, as the gastrocnemius muscle is stretched to nearly its longest length in bottom position [9], we detected the bottom position by measuring the peak of air pressure in a gastrocnemius muscle (P_{gas}) (described by the following equation).

$$\frac{\partial P_{gas}}{\partial t} = 0$$

Based on the data of human vertical hopping and jumping EMG [29] [30] [31] and by considering the past jumping robot control [57] [58], we generated the control sequence consisting of three states: Flying, Landing, and Pushing Off. The control program runs in a loop (shown in Fig. 2) and explained as follows:

1) Flying State: This is the initialization state for the preparation of the landing. The robot adjusts the air in each muscle to a predetermined initialization pressure. The next state is activited after the first foot touches the ground.

2) Landing State: In this state, except the soleus mucles used to mimic stretch reflex, the robot closes all the valves. Stretch reflex is applied in this state. We applied and tested different reflexive controls on the soleus muscles (for stretch reflex). This state terminates when the robot reaches the bottom posture in squat.

3) Pushing Off State: Compressed air is supplied to gluteus maximus, vastus lateralis, and soleus muscles. Upward thrust could be generated to drive the robot lifting off.

Appendix D
Other Devices

FSRs (FSR-406, Interlink Electronics) with a voltage divider of 7.5 $k\Omega$ were used to detect the touchdown and start of muscle stretching. The compressed air was controlled using pneumatic valves (PSE540, SMC Corporation) and generated by a compressor (2000-40m, Jun Air) through a tether. To measure the air pressure in the PAMs, we used the pressure sensors (PSE530) produced by SMC Corporation. A gyroscope (CRS03, Silicon Sensing Systems) and a three-axis accelerometer (KXR94, Kionix) were combined in a complementary filter to obtain the lateral inclination.

The complementary filter estimated the lateral inclination by using the one-axis data from gyroscope and two-axis data from the accelerometer. First, the accelerometer information of both Y and Z axes were used to find the angular projection in the frontal plane:

$$\theta_{a(x,n)} = arctan(\frac{y}{z}),$$

where y and z represent the acceleration information in Y and Z axes respectively. $\theta_{(x,n)}$ is the lateral inclination (about X axis) calculated by the accelerometer.

The accelerometer shows fast reaction and large noises, whereas the gyroscope has a more stable output but with larger delay. We estimated the body lateral inclination by combining the calculation of accelerometer and gyroscope. The estimated lateral inclination is presented as follows:

$$\theta_{e(x,n)} = \frac{\theta_{a(x,n)} + w\theta_{g(x,n)}}{1+w},$$

where w is the filter weight set as 90 in this experiment and $\theta_{g(x,n)}$ is the lateral inclination output of the gyroscope.

Considering that the accelerometer registers angular velocity, the gyroscope used a previous angular estimation to update itself:

$$\theta_{g(x,n)} = \theta_{e(x,n-1)} + \dot{\theta}_{g(x,n)} T$$

where T is the sampling duration.

References

[1] Bouvier J, Caggiano V, Leiras R, Caldeira V, Bellardita C, Balueva K, et al. Descending command neurons in the brainstem that halt locomotion. Cell. 2015;163(5):1191–1203. Elsevier.

[2] Mezzarane RA, Nakajima T, Zehr EP. After stroke bidirectional modulation of soleus stretch reflex amplitude emerges during rhythmic arm cycling. Frontiers in human neuroscience. 2014;8:136. Frontiers. doi:10.3389/fnhum.2014.00136.

[3] Stubbs PW, Nielsen JF, Sinkjær T, Mrachacz-Kersting N. Short-latency crossed spinal responses are impaired differently in sub-acute and chronic stroke patients. Clinical Neurophysiology. 2012;123(3):541–549. Elsevier.

[4] Takeoka A, Vollenweider I, Courtine G, Arber S. Muscle spindle feedback directs locomotor recovery and circuit reorganization after spinal cord injury. Cell. 2014;159(7):1626–1639. Elsevier.

[5] Pearson K. Could enhanced reflex function contribute to improving locomotion after spinal cord repair? The Journal of physiology. 2001;533(1):75–81. Wiley Online Library. doi:10.1111/j.1469-7793.2001.0075b.x.

[6] Ijspeert AJ. Biorobotics: Using robots to emulate and investigate agile locomotion. Science. 2014;346(6206):196–203. American Association for the Advancement of Science. doi:10.1126/science.1254486.

[7] Floreano D, Ijspeert AJ, Schaal S. Robotics and neuroscience. Current Biology. 2014;24(18):R910–R920. Elsevier. doi:10.1016/j.cub.2014.07.058.

[8] Zuur AT, Lundbye-Jensen J, Leukel C, Taube W, Grey MJ, Gollhofer A, et al. Contribution of afferent feedback and descending drive to human hopping. The Journal of physiology. 2010;588(5):799–807. Wiley Online Library. doi:10.1113/jphysiol.2009.182709.

[9] Voigt M, Dyhre-Poulsen P, Simonsen E. Modulation of short latency stretch reflexes during human hopping. Acta Physiologica Scandinavica. 1998;163(2):181–194. Wiley Online Library. doi:10.1046/j.1365-201X.1998.00351.x.

[10] Geyer H, Herr H. A muscle-reflex model that encodes principles of legged mechanics produces human walking dynamics and muscle activities. IEEE Transactions on neural systems and rehabilitation engineering. 2010;18(3):263–273. IEEE. doi:10.1109/TNSRE.2010.2047592.

[11] Van der Noot N, Ijspeert AJ, Ronsse R. Biped gait controller for large speed variations, combining reflexes and a central pattern generator in a neuromuscular model. In: Robotics and Automation (ICRA), 2015 IEEE International Conference on. IEEE; 2015. p. 6267–6274. doi:10.1109/ICRA.2015.7140079.

[12] Taga G. A model of the neuro-musculo-skeletal system for human locomotion. Biological cybernetics. 1995;73(2):97–111. Springer. doi:https://doi.org/10.1007/BF00204048.

[13] Nicol C, Komi PV. Significance of passively induced stretch reflexes on achilles tendon force enhancement. Muscle & nerve. 1998;21(11):1546–1548. doi:10.1002/(SICI)1097-4598(199811)21:11¡1546::AID-MUS29¿3.0.CO;2-X.

[14] Ogawa T, Kawashima N, Suzuki S, Nakazawa K. Different modulation pattern of spinal stretch reflex excitability in highly trained endurance runners. European journal of applied physiology. 2012;112(10):3641–3648. Springer. doi:https://doi.org/10.1007/s00421-012-2351-7.

[15] Capaday C, Stein R. Difference in the amplitude of the human soleus H reflex during walking and running. The Journal of physiology. 1987;392:513. Wiley-Blackwell. doi:10.1113/jphysiol.1987.sp016794.

[16] McIllroy W, Collins D, Brooke J. Movement features and H-reflex modulation. II. Passive rotation, movement velocity and single leg movement. Brain research. 1992;582(1):85–93. Elsevier. doi:https://doi.org/10.1016/0006-8993(92)90320-9.

[17] Cheng J, Brooke J, Misiaszek J, Staines W. The relationship between the kinematics of passive movement, the stretch of extensor muscles of the leg and the change induced in the gain of the soleus H reflex in humans. Brain research. 1995;672(1):89–96. Elsevier. doi:https://doi.org/10.1016/0006-8993(94)01321-8.

[18] Stubbs PW, Mrachacz-Kersting N. Short-latency crossed inhibitory responses in the human soleus muscle. Journal of neurophysiology. 2009;102(6):3596–3605. Am Physiological Soc. doi::10.1152/jn.00667.2009.

[19] Stubbs PW, Nielsen JF, Sinkjær T, Mrachacz-Kersting N. Crossed spinal soleus muscle communication demonstrated by H-reflex conditioning. Muscle & nerve. 2011;43(6):845–850. Wiley Online Library. doi:10.1002/mus.21964.

[20] Gervasio S, Voigt M, Kersting UG, Farina D, Sinkjær T, Mrachacz-Kersting N. Sensory Feedback in Interlimb Coordination: Contralateral Afferent Contribution to the Short-Latency Crossed Response during Human Walking. PloS one. 2017;12(1):e0168557. Public Library of Science.

[21] Gervasio S, Farina D, Sinkjær T, Mrachacz-Kersting N. Crossed reflex reversal during human locomotion. Journal of neurophysiology. 2013;109(9):2335–2344. Am Physiological Soc. doi:10.1152/jn.01086.2012.

[22] Stubbs PW, Nielsen JF, Sinkjær T, Mrachacz-Kersting N. Phase modulation of the short-latency crossed spinal response in the human soleus muscle. Journal of neurophysiology. 2011;105(2):503–511. Am Physiological Soc. doi:10.1152/jn.00786.2010.

[23] Gervasio S, Kersting UG, Farina D, Mrachacz-Kersting N. The effect of crossed reflex responses on dynamic stability during locomotion. Journal of neurophysiology. 2015;114(2):1034–1040. Am Physiological Soc. doi:10.1152/jn.00178.2015.

[24] Ramdya P, Thandiackal R, Cherney R, Asselborn T, Benton R, Ijspeert AJ, et al. Climbing favours the tripod gait over alternative faster insect gaits. Nature communications. 2017;8. Nature Publishing Group.

[25] Manoonpong P, Petersen D, Kovalev A, Wörgötter F, Gorb SN, Spinner M, et al. Enhanced Locomotion Efficiency of a Bio-inspired Walking Robot using Contact Surfaces with Frictional Anisotropy. Scientific reports. 2016;6:39455. Nature Publishing Group. doi:10.1038/srep39455.

[26] Kawabata K, Aonuma H, Hosoda K, Sugimoto Y, Xue J. Experimental study on robotic interactions to the cricket. In: Robotics and Biomimetics (ROBIO), 2014 IEEE International Conference on. IEEE; 2014. p. 949–954. doi:10.1109/ROBIO.2014.7090455.

[27] Spröwitz A, Tuleu A, Vespignani M, Ajallooeian M, Badri E, Ijspeert AJ. Towards dynamic trot gait locomotion: Design, control, and experiments with Cheetah-cub, a compliant quadruped robot. The International Journal of Robotics Research. 2013;32(8):932–950. SAGE Publications Sage UK: London, England. doi:10.1177/0278364913489205.

[28] Hobara H, Kanosue K, Suzuki S. Changes in muscle activity with increase in leg stiffness during hopping. Neuroscience letters. 2007;418(1):55–59. Elsevier. doi:https://doi.org/10.1016/j.neulet.2007.02.064.

[29] Bobbert MF, van Ingen Schenau GJ. Coordination in vertical jumping. Journal of biomechanics. 1988;21(3):249–262. Elsevier. doi:http://dx.doi.org/10.1016/0021-9290(88)90175-3.

[30] Viitasalo J, Bosco C. Electromechanical behaviour of human muscles in vertical jumps. European journal of applied physiology and occupational physiology. 1982;48(2):253–261. Springer. doi:https://doi.org/10.1007/BF00422986.

[31] Pandy MG, Zajac FE. Optimal muscular coordination strategies for jumping. Journal of biomechanics. 1991;24(1):1–10. Elsevier.

[32] Kothera CS, Philen M, Tondu B. Modelling of the McKibben artificial muscle: A review. Journal of Intelligent Material Systems and Structures. 2012;23(3):225–253. Sage Publications Sage UK: London, England. doi:10.1177/1045389X11435435.

[33] Hay JG, Reid JG. Anatomy, mechanics, and human motion. Prentice Hall; 1988.

[34] Farris DJ, Sawicki GS. Linking the mechanics and energetics of hopping with elastic ankle exoskeletons. Journal of Applied Physiology. 2012;113(12):1862–1872. Am Physiological Soc. doi:10.1152/japplphysiol.00802.2012.

[35] van der Krogt MM, de Graaf WW, Farley CT, Moritz CT, Casius LR, Bobbert MF. Robust passive dynamics of the musculoskeletal system compensate for unexpected surface changes during human hopping. Journal of Applied Physiology. 2009;107(3):801–808. Am Physiological Soc. doi:10.1152/japplphysiol.91189.2008.

[36] Hanna-Boutros B, Sangari S, Karasu A, Giboin LS, Marchand-Pauvert V. Task-related modulation of crossed spinal inhibition between human lower limbs. Journal of neurophysiology. 2014;111(9):1865–1876. Am Physiological Soc. doi:10.1152/jn.00838.2013.

[37] Dietz V, Noth J. Pre-innervation and stretch responses of triceps bracchii in man falling with and without visual control. Brain research. 1978;142(3):576–579. Elsevier.

[38] Kuitunen S, Ogiso K, Komi P. Leg and joint stiffness in human hopping. Scandinavian journal of medicine & science in Sports. 2011;21(6):e159–e167. Wiley Online Library. doi:10.1111/j.1600-0838.2010.01202.x.

[39] Stevenson AJ, Geertsen SS, Andersen JB, Sinkjær T, Nielsen JB, Mrachacz-Kersting N. Interlimb communication to the knee flexors during walking in humans. The Journal of physiology. 2013;591(19):4921–4935. Wiley Online Library. doi:10.1113/jphysiol.2013.257949.

[40] Gervasio S. Interlimb communication during human walking: Crossed responses in the gastrocnemius muscle. River Publishers; 2014.

[41] Fong SS, Guo X, Liu KP, Ki W, Louie LH, Chung RC, et al. Task-specific balance training improves the sensory organisation of balance control in children with developmental coordination disorder: a randomised controlled trial. Scientific reports. 2016;6. Nature Publishing Group.

[42] Haeufle D, Grimmer S, Kalveram KT, Seyfarth A. Integration of intrinsic muscle properties, feed-forward and feedback signals for generating and stabilizing hopping. Journal of The Royal Society Interface. 2012;9(72):1458–1469. The Royal Society. doi:10.1098/rsif.2011.0694.

[43] Geyer H, Seyfarth A, Blickhan R. Positive force feedback in bouncing gaits? Proceedings of the Royal Society of London B: Biological Sciences. 2003;270(1529):2173–2183. The Royal Society. doi:10.1098/rspb.2003.2454.

[44] Cheng J, Brooke JD, Misiaszek JE, Staines WR. Crossed inhibition of the soleus H reflex during passive pedalling movement. Brain research. 1998;779(1):280–284. Elsevier. doi:https://doi.org/10.1016/S0006-8993(97)01168-2.

[45] Suzuki S, Nakajima T, Futatsubashi G, Mezzarane RA, Ohtsuka H, Ohki Y, et al. Soleus Hoffmann reflex amplitudes are specifically modulated by cutaneous inputs from the arms and opposite leg during walking but not standing. Experimental brain research. 2016;p. 1–12. Springer. doi:10.1007/s00221-016-4635-3.

[46] Suzuki S, Nakajima T, Mezzarane RA, Ohtsuka H, Futatsubashi G, Komiyama T. Differential regulation of crossed cutaneous effects on the soleus H-reflex during standing and walking in humans. Experimental brain research. 2014;232(10):3069–3078. Springer. doi:10.1007/s00221-014-3953-6.

[47] Knikou M, Rymer WZ. Static and dynamic changes in body orientation modulate spinal reflex excitability in humans. Experimental brain research. 2003;152(4):466–475. Springer.

[48] Davis JR, Horslen BC, Nishikawa K, Fukushima K, Chua R, Inglis JT, et al. Human proprioceptive adaptations during states of height-induced fear and anxiety. Journal of neurophysiology. 2011;106(6):3082–3090. Am Physiological Soc. doi:10.1152/jn.01030.2010.

[49] Horslen BC, Murnaghan CD, Inglis JT, Chua R, Carpenter MG. Effects of postural threat on spinal stretch reflexes: evidence for increased muscle spindle sensitivity? Journal of neurophysiology. 2013;110(4):899–906. Am Physiological Soc. doi:10.1152/jn.00065.2013.

[50] Phanthanourak AL, Cleworth TW, Adkin AL, Carpenter MG, Tokuno CD. The threat of a support surface translation affects anticipatory postural control. Gait & posture. 2016;50:145–150. Elsevier. doi:https://doi.org/10.1016/j.gaitpost.2016.08.031.

[51] Dickinson MH, Farley CT, Full RJ, Koehl M, Kram R, Lehman S. How animals move: an integrative view. Science. 2000;288(5463):100–106. American Association for the Advancement of Science. doi:10.1126/science.288.5463.100.

[52] Klute GK, Czerniecki JM, Hannaford B. McKibben artificial muscles: pneumatic actuators with biomechanical intelligence. In: Advanced Intelligent Mechatronics, 1999. Proceedings. 1999 IEEE/ASME International Conference on. IEEE; 1999. p. 221–226. doi:10.1109/AIM.1999.803170.

[53] Shin H, Saitoh H, Kawakami T, Yamanishi S, Ikemoto S, Hosoda K. Development of an embedded sensor system for pneumatic artificial muscle proprioceptors. Artificial Life and Robotics. 2016;21(4):486–492. Springer. doi:10.1007/s10015-016-0290-9.

[54] van Ingen Schenau Gv, Bobbert M, Rozendal R. The unique action of bi-articular muscles in complex movements. Journal of Anatomy. 1987;155:1. Wiley-Blackwell.

[55] Kumamoto M, Oshima T, Yamamoto T. Control properties induced by the existence of antagonistic pairs of bi-articular musclesłechanical engineering model analyses. Human Movement Science. 1994;13(5):611–634. Elsevier.

[56] Nagano A, Komura T, Fukashiro S, Himeno R. Force, work and power output of lower limb muscles during human maximal-effort countermovement jumping. Journal of Electromyography and Kinesiology. 2005;15(4):367–376. Elsevier. doi:10.1016/j.jelekin.2004.12.006.

[57] Hosoda K, Sakaguchi Y, Takayama H, Takuma T. Pneumatic-driven jumping robot with anthropomorphic muscular skeleton structure. Au-

tonomous Robots. 2010;28(3):307–316. Springer. doi:10.1007/s10514-009-9171-6.
[58] Raibert MH. Legged robots that balance. MIT press; 1986.
