# Supplementary material for: Robotic investigation on effect of stretch reflex and crossed inhibitory response on bipedal hopping
Source: J R Soc Interface. 2018 Mar 28;15(140):20180024. doi: 10.1098/rsif.2018.0024 (PMC5908536; doi:10.1098/rsif.2018.0024)
Supplement: Response to Referees [file rsif20180024supp1.docx]

Authors’ Responses

*Response to the 2nd Reviewer*

*I commend the authors for the taking the time to carefully address the reviewer's comments. The manuscript has greatly improved. I have no further comments regarding the scientific merit of the manuscript.*

We thank the reviewer for the kind comments.

*However, I do encourage the authors to seek further assistance in re-wording several parts in the manuscript, which I feel can help improve its readability.*

We sought advice from Editage ltd. (an English re-wording company recommended by the Royal Society Publication) and improved the quality of the English expressions. Moreover, our group discussed the output from Editage and made additional efforts to improve the quality of the scientific writing. We believe that the revised manuscript has a better quality and readability. Moreover, detailed information about re-wording can be found in attached file (Rewording.pdf).

Response to the 1st Reviewer

*This new version of the paper is significantly improved. The paper better conveys the message and some of the technical issues were solved.*

We thank the reviewer for the kind comments.

*I still think that centre of mass (CoM) tracks are required, both to bake the assumption on lateral stability and also to provide a comparison for further studies. I am sure that authors have several videos, and such shift on the CoM should not be difficult to obtain and plot, similarly to Fig5. My suggestion is to add these details which will be relevant for readers to understand the whole picture of the behaviour, and which will allow more accurate replications of your study by other authors.*

Please allow us to explain the issue of COM:

We appreciate the suggestion and we have carefully considered to record the centre of mass (COM) during the experiments. Assuming that the mass of the leg is not negligible with respect to that of the body, the location of the COM moves when the robot extends/contracts the legs (shown in Fig. 1). Therefore, it is very difficult to estimate the location of COM precisely from the recorded image/video during dynamic locomotion. If we approximate the robot as a lumped mass neglecting the mass of the legs, the difference in motions of the robot may be small as that in the angles. Although we may be able to observe small differences using force sensors, this observation would be very noisy, as force sensors are prone to disturbances associated to the impact against the floor during touchdown of landing.

Fig. 1 The COM moves upward and forward as the compress of the legs during hopping

In our experiments, we recorded θ (lateral inclination) to evaluate the change of lateral posture. θ has a direct connection with the COM (shown in Fig. 2), as a smaller displacement/movement of the COM results in the decrement of θ. Similar approaches are widely used in experiments on both humans and robots.

Fig. 2 A smaller displacement/movement of centre of mass (COM) indicates the decrement of lateral inclination (θ)
